# The Relationship Between Tri-ponderal Mass Index and Metabolic Syndrome and Its Components in Youth Aged 10–20 Years

**DOI:** 10.1038/s41598-019-50987-3

**Published:** 2019-10-08

**Authors:** Young Suk Shim

**Affiliations:** Department of Pediatrics, Hallym University Dongtan Sacred Heart Hospital, Hwaseong, Korea

**Keywords:** Obesity, Metabolic syndrome

## Abstract

The current study aimed to evaluate the distribution of the tri-ponderal mass index (TMI) according to sex and age and the relationship of obesity groups according to sex- and age-specific TMI with metabolic syndrome (MetS) and its components. A total of 8,464 subjects aged 10–20 years were classified into 4 groups according to sex- and age-specific TMI: (i) underweight, (ii) normal weight, (iii) overweight, and (iv) obese. The range of the 50th percentiles of TMI was from 13.24 kg/m^3^ at 10 years to 12.94 kg/m^3^ at 20 years among males and from 12.19 kg/m^3^ to 12.84 kg/m^3^ among females. In the analysis of covariance, obesity groups according to sex- and age-specific TMI were positively correlated with waist circumference (WC) standard deviation score; systolic blood pressure (BP); diastolic BP; and levels of glucose, total cholesterol, triglycerides (TGs), and low-density lipoprotein cholesterol, but for both sexes, the obesity groups were negatively related to high-density lipoprotein cholesterol (HDL-C). In the multiple logistic regression, subjects in the overweight group had higher odds ratios (ORs) for elevated WC (29.18), elevated BP (1.33), elevated TGs (2.55), reduced HDL-C (2.31), and MetS (8.93) than those with normal weight. Participants in the obesity group had increased ORs for elevated WC (154.67), elevated BP (2.22), elevated glucose (3.54), elevated TGs (4.12), reduced HDL-C (3.69), and MetS (25.57) compared to participants with normal weight after adjustment for confounders. Our results suggest that sex- and age-specific TMI may be applicable in the clinical setting as a useful screening tool.

## Introduction

With the increasing prevalence of childhood and adolescent obesity worldwide, including in the republic of Korea^1,2^, a large international concern has developed in the medical community regarding children with excess weight who are at an increased risk for cardiometabolic risk factors, including abdominal obesity, impaired glucose metabolism, insulin resistance, hypertension, and hypercholesterolemia^3^. Childhood and adolescent metabolic syndrome (MetS), which is defined as the clustering of elevated waist circumference (WC), elevated blood pressure (BP), elevated glucose level, and dyslipidemia (elevated levels of triglycerides (TGs) and/or reduced levels of high-density lipoprotein cholesterol (HDL-C)), begins as early as childhood^4^ and tends to persist from the childhood period into adulthood^5^. Childhood MetS is a risk factor for MetS, cardiovascular diseases and type 2 diabetes mellitus (T2DM) in adulthood^6^. Because this constellation of cardiometabolic risk factors could be modifiable, early identification of children and adolescents with MetS is essential to improve their condition via age-specific interventions.

To date, body mass index (BMI), which is the ratio of body weight to height squared (kg/m^2^), has been used for the classification of excess body fat in children, adolescents and adults. Because weight is not proportional to the square of height during the adolescent period that is characterized by rapid growth and development^7^, the validity of BMI values is weakened. Instead of BMI values, the standard deviation score (SDS) of BMI is used for the classification of obesity among children and adolescents. However, this approach is considered to have difficulties taking into account that both body proportions and body fat levels change during the adolescent growth period. A recent study suggested that the tri-ponderal mass index (TMI), which is the ratio of body weight to height cubed (kg/m^3^), is apparently stable during adolescence and could estimate body fat levels more accurately than BMI, especially in children and adolescents^8^. Some studies have reported that TMI is a better predictor of cardiometabolic risk than BMI^9,10^. However, no studies have reported the prevalence of MetS and its components according to TMI in children, adolescents, and young adults.

In the current study, we aimed to evaluate the distribution of TMI according to sex and age and the relationship between obesity groups according to sex- and age-specific TMI and MetS and its components, which are considered a surrogate for cardiometabolic risk factors, using nationally representative data in youth aged 10–20 years.

## Results

### Clinical characteristics of the study population

The clinical characteristics of the study population are shown in Table 1. The boys and men in the obesity group according to sex and age-specific TMI had a higher mean weight standard deviation score (SDS) (*P* < 0.001), WC SDS (*P* < 0.001), BMI SDS (*P* < 0.001), TMI SDS (*P* < 0.001), systolic BP (SBP) (*P* < 0.001), diastolic BP (DBP) (*P* < 0.001), glucose level (*P* < 0.001), total cholesterol (T-C) level (*P* < 0.001), TG level (*P* < 0.001), and low-density lipoprotein cholesterol (LDL-C) level (*P* < 0.001), whereas they had a lower mean HDL-C level (*P* < 0.001). The male subjects in the obesity group according to sex- and age-specific TMI tended to have hypertension (*P* = 0.030), diabetes mellitus (DM) (*P* < 0.001), and dyslipidemia (*P* < 0.001). The girls and women in the obesity group according to sex and age-specific TMI had a higher mean weight SDS (*P* < 0.001), WC SDS (*P* < 0.001), BMI SDS (*P* < 0.001), TMI SDS (*P* < 0.001), SBP (*P* < 0.001), DBP (*P* < 0.001), glucose level (*P* < 0.001), T-C level (*P* < 0.001), TG level (*P* < 0.001), and LDL-C level (*P* < 0.001), whereas they had a lower mean height SDS (*P* < 0.001) and HDL-C level (*P* < 0.001). The female subjects in the obesity group according to sex- and age-specific TMI tended to have DM (*P* < 0.001).

**Table 1:** Clinical characteristics of the study population (*n* = 8,464).

Boys and men					
	TMI group				
	Underweight	Normal weight	Overweight	Obesity	*P*
	*n* = 140	*n* = 3577	*n* = 441	*n* = 213	
Age (years)	14.06 ± 2.84	14.32 ± 2.89	14.28 ± 2.90	14.31 ± 2.87	0.779
Height SDS	1.00 ± 1.03	0.73 ± 1.07	0.73 ± 1.12	0.73 ± 1.07	0.266
Weight SDS	−1.12 ± 0.96	0.23 ± 0.88	1.59 ± 0.58	2.23 ± 0.59	<0.001
WC SDS	−1.99 ± 0.96	−0.36 ± 1.07	1.23 ± 0.45	1.78 ± 0.51	<0.001
BMI SDS	−2.08 ± 0.68	−0.09 ± 0.82	1.53 ± 0.29	2.20 ± 0.37	<0.001
TMI (kg/m^2^)	9.35 ± 0.45	12.28 ± 1.45	15.95 ± 0.68	18.49 ± 1.48	<0.001
SBP (mmHg)	103.42 ± 8.96	108.33 ± 10.45	114.00 ± 10.05	117.48 ± 12.11	<0.001
DBP (mmHg)	66.06 ± 9.88	66.73 ± 9.53	68.51 ± 9.68	71.00 ± 10.47	<0.001
Glucose (mg/dL)	89.41 ± 5.36	90.09 ± 7.25	91.29 ± 6.60	92.71 ± 7.67	<0.001
T-C (mg/dL)	151.07 ± 22.56	154.01 ± 25.88	167.69 ± 30.72	171.21 ± 30.66	<0.001
TGs	74.96 ± 42.89	80.68 ± 49.08	109.14 ± 63.58	123.41 ± 70.27	<0.001
HDL-C	53.01 ± 10.41	50.42 ± 9.79	45.56 ± 8.99	43.84 ± 8.17	<0.001
LDL-C	83.01 ± 18.42	87.41 ± 21.86	100.22 ± 27.77	102.64 ± 27.55	<0.001
Alcohol consumption (%)	11 (7.9%)	399 (11.2%)	41 (9.3%)	20 (9.4%)	0.361
Smoking (%)	14 (10.0%)	522 (14.6%)	56 (12.7%)	31 (14.6%)	0.348
Physical activity (%)	54 (38.6%)	1542 (43.4%)	182 (41.3%)	84 (39.4%)	0.412
Household income <2nd percentile (%)	21 (15.0%)	400 (11.2%)	49 (11.1%)	27 (12.7%)	0.504
Rural residence (%)	18 (12.9%)	590 (16.5%)	64 (14.5%)	39 (18.3%)	0.395
Hypertension (%)	0 (0%)	4 (0.1%)	1 (0.2%)	2 (0.9%)	0.030
Diabetes mellitus (%)	0 (0%)	3 (0.1%)	1 (0.2%)	4 (1.9%)	<0.001
Dyslipidemia (%)	0 (0%)	0 (0%)	0 (0%)	1 (0.5%)	<0.001
**Girls and women**					
	**TMI group**				
	**Underweight**	**Normal weight**	**Overweight**	**Obesity**	***P***
	***n*** = **116**	***n*** = **3364**	***n*** = **413**	***n*** = **200**	
Age (years)	14.66 ± 3.07	14.72 ± 3.08	14.70 ± 3.08	14.75 ± 3.08	0.885
Height SDS	1.10 ± 0.87	0.48 ± 1.03	0.37 ± 1.15	0.18 ± 1.25	<0.001
Weight SDS	−1.28 ± 0.86	0.05 ± 0.91	1.44 ± 0.69	2.08 ± 0.76	<0.001
WC SDS	−1.82 ± 0.78	−0.36 ± 0.93	1.07 ± 0.68	1.75 ± 0.71	<0.001
BMI SDS	−2.26 ± 0.68	−0.17 ± 0.81	1.50 ± 0.33	2.25 ± 0.45	<0.001
TMI (kg/m^2^)	9.52 ± 0.44	12.39 ± 1.26	15.73 ± 0.73	18.19 ± 1.55	<0.001
SBP (mmHg)	101.10 ± 9.26	103.46 ± 8.93	106.98 ± 9.59	110.48 ± 10.88	<0.001
DBP (mmHg)	65.76 ± 8.02	65.68 ± 8.27	66.23 ± 8.30	69.20 ± 9.4	<0.001
Glucose (mg/dL)	89.53 ± 7.58	88.46 ± 7.79	89.54 ± 6.67	92.47 ± 12.86	<0.001
T-C (mg/dL)	158.86 ± 25.08	162.99 ± 26.05	168.40 ± 27.61	171.14 ± 29.90	<0.001
TGs	76.96 ± 36.64	82.17 ± 44.43	102.60 ± 68.45	118.19 ± 68.46	<0.001
HDL-C	55.52 ± 9.22	53.43 ± 10.13	48.60 ± 8.78	45.67 ± 9.18	<0.001
LDL-C	87.95 ± 21.96	93.08 ± 22.62	99.23 ± 25.43	101.75 ± 27.45	<0.001
Alcohol consumption (%)	9 (7.8%)	340 (10.1%)	39 (9.4%)	17 (8.5%)	0.730
Smoking (%)	11 (9.48%)	181 (5.38%)	28 (6.78%)	14 (7.00%)	0.155
Physical activity (%)	35 (30.2%)	1080 (32.1%)	121 (29.3%)	57 (28.5%)	0.492
Household income <2nd percentile (%)	11 (9.5%)	376 (11.2%)	58 (14.0%)	31 (15.5%)	0.092
Rural residence (%)	18 (15.5%)	516 (15.3%)	70 (17.0%)	36 (18.0%)	0.656
Hypertension (%)	0 (0%)	0 (0%)	0 (0%)	0 (0%)	>0.999
Diabetes mellitus (%)	1 (0.9%)	4 (0.1%)	0 (0%)	6 (3.0%)	<0.001
Dyslipidemia (%)	0 (0%)	0 (0%)	0 (0%)	0 (0%)	>0.999

Data are presented as the mean ± standard deviation (SD).

SDS, standard deviation score; WC, waist circumference; BMI, body mass index; TMI, tri-ponderal mass index; SBP, systolic blood pressure; DBP, diastolic blood pressure; T-C, total cholesterol; HDL-C, high-density lipoprotein cholesterol; TGs, triglycerides; LDL-C, low-density lipoprotein cholesterol.

### Distribution of TMI according to sex and age

**Figure 1:**
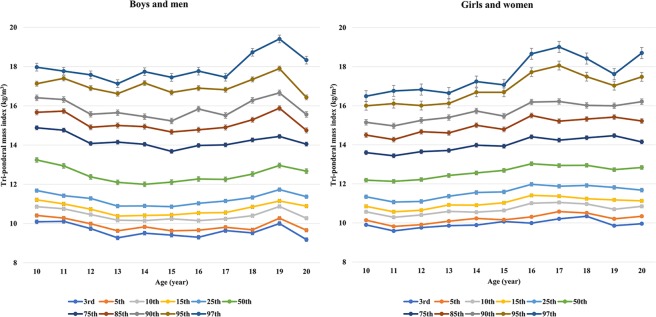
Distribution of tri-ponderal mass index (TMI) according to sex and age.

The distribution of TMI based on sex and age are presented in Table 2 and Fig. 1. The 50th percentile values of TMI were apparently stable during childhood and adolescence. The range of the TMI values was from 13.24 kg/m^3^ at the age of 10 years to 12.94 kg/m^3^ at the age of 20 years among boys and men (U-shaped) and from 12.19 kg/m^3^ to 12.84 kg/m^3^ among girls and women (inverted U-shaped).

**Table 2:** Distribution of tri-ponderal mass index (TMI) according to sex and age (*n* = 8,464).

Boys and men														
Age	n	mean	SD	Percentile										
				3rd	5th	10th	15th	25th	50th	75th	85th	90th	95th	97th
10	439	13.42	2.18	10.09	10.41	10.86	11.21	11.68	13.24	14.88	15.67	16.41	17.13	17.97
11	483	13.26	2.29	10.11	10.28	10.75	11.00	11.42	12.94	14.76	15.73	16.32	17.40	17.77
12	483	12.78	2.08	9.74	9.99	10.46	10.73	11.28	12.38	14.08	14.91	15.57	16.90	17.58
13	494	12.56	2.23	9.27	9.63	10.17	10.38	10.89	12.10	14.15	15.00	15.65	16.62	17.13
14	493	12.59	2.27	9.51	9.83	10.15	10.41	10.90	12.00	14.04	14.94	15.45	17.16	17.74
15	442	12.49	2.18	9.41	9.63	10.24	10.44	10.86	12.11	13.68	14.67	15.23	16.68	17.45
16	381	12.66	2.22	9.30	9.66	10.15	10.54	11.03	12.27	13.98	14.78	15.84	16.90	17.77
17	383	12.65	2.11	9.64	9.81	10.24	10.56	11.15	12.25	14.01	14.90	15.51	16.82	17.46
18	355	12.99	2.36	9.52	9.68	10.40	10.85	11.33	12.52	14.26	15.29	16.28	17.35	18.73
19	289	13.41	2.43	10.00	10.27	10.87	11.15	11.73	12.96	14.44	15.88	16.66	17.90	19.41
20	129	12.85	2.14	9.18	9.66	10.27	10.89	11.37	12.67	14.05	14.75	15.56	16.43	18.33
Total	4371	12.86	2.25	9.62	9.90	10.33	10.68	11.22	12.45	14.22	15.17	15.92	16.99	17.76
**Girls and women**														
**Age**	**n**	**mean**	**SD**	**Percentile**										
				**3rd**	**5th**	**10th**	**15th**	**25th**	**50th**	**75th**	**85th**	**90th**	**95th**	**97th**
10	377	12.55	1.76	9.90	10.14	10.57	10.85	11.34	12.19	13.60	14.50	15.15	16.00	16.49
11	415	12.44	1.92	9.59	9.82	10.29	10.57	11.07	12.13	13.44	14.27	14.97	16.11	16.76
12	399	12.51	1.95	9.76	9.91	10.41	10.65	11.10	12.22	13.65	14.67	15.25	16.01	16.83
13	420	12.71	1.84	9.86	10.09	10.59	10.92	11.37	12.43	13.71	14.61	15.40	16.12	16.65
14	437	12.91	2.05	9.89	10.23	10.55	10.91	11.56	12.56	13.98	15.00	15.73	16.69	17.24
15	362	12.91	1.92	10.07	10.16	10.64	11.03	11.59	12.69	13.93	14.79	15.47	16.69	17.07
16	372	13.40	2.24	9.99	10.31	11.01	11.41	11.98	13.03	14.41	15.50	16.19	17.72	18.66
17	380	13.33	2.18	10.21	10.58	11.05	11.37	11.88	12.94	14.24	15.21	16.22	18.06	19.01
18	302	13.33	2.09	10.34	10.51	10.97	11.24	11.92	12.95	14.36	15.32	16.02	17.49	18.42
19	330	13.18	2.14	9.86	10.21	10.70	11.18	11.82	12.73	14.47	15.42	16.00	17.03	17.62
20	299	13.20	2.17	9.96	10.34	10.85	11.13	11.68	12.84	14.15	15.22	16.21	17.48	18.70
Total	4093	12.93	2.05	9.92	10.16	10.63	10.98	11.52	12.62	13.97	14.92	15.67	16.78	17.58

SD, standard deviation.

### Adjusted means of cardiometabolic risk factors according to sex- and age-specific TMI obesity groups

The adjusted means of cardiometabolic risk factors, including WC SDS; SBP; DBP; and levels of glucose, T-C, HDL-C, TGs, and LDL-C, are presented in Table 3. In both sexes, a significant positive correlation was observed between the obesity groups categorized according to sex- and age-specific TMI and WC SDS (*P* < 0.001 for trend); SBP (*P* < 0.001 for trend); DBP (*P* < 0.001 for trend); and the levels of glucose, T-C, TGs, and LDL-C, whereas a significant negative correlation was found between the obesity groups categorized according to sex- and age-specific TMI and HDL-C levels (*P* < 0.001 for trend) after adjusting for possible confounders using an analysis of covariance (ANCOVA).

**Table 3:** Adjusted mean values of cardiometabolic risk factors according to sex- and age-specific tri-ponderal mass index (TMI) groups (*n* = 8,464).

Boys and men					
	TMI group				
	Underweight	Normal weight	Overweight	Obesity	*P*
WC SDS	−1.98 ± 0.09	−0.36 ± 0.02^a^	1.23 ± 0.05^a,b^	1.76 ± 0.07^a,b,c^	<0.001
SBP (mmHg)	103.70 ± 0.83	108.33 ± 0.16^a^	114.00 ± 0.47^a,b^	117.31 ± 0.68^a,b,c^	<0.001
DBP (mmHg)	66.35 ± 0.74	66.72 ± 0.15	68.51 ± 0.42^b^	70.88 ± 0.61^a,b,c^	<0.001
Glucose (mg/dL)	89.35 ± 0.57	90.15 ± 0.11	91.26 ± 0.32^a,b^	91.93 ± 0.47^a,b^	<0.001
T-C (mg/dL)	150.86 ± 2.22	154.03 ± 0.44	167.70 ± 1.25^a,b^	170.87 ± 1.81^a,b^	<0.001
TGs	75.51 ± 4.36	80.69 ± 0.86	109.20 ± 2.45^a,b^	122.62 ± 3.55^a,b,c^	<0.001
HDL-C	52.87 ± 0.80	50.41 ± 0.16^a^	45.58 ± 0.45^a,b^	44.01 ± 0.65^a,b^	<0.001
LDL-C	82.85 ± 1.91	87.44 ± 0.38	100.20 ± 1.08^a,b^	102.29 ± 1.56^a,b^	<0.001
**Girls and women**					
	**Underweight**	**Normal weight**	**Overweight**	**Obesity**	***P***
WC SDS	−1.82 ± 0.08	−0.36 ± 0.02	1.07 ± 0.04^a,b^	1.74 ± 0.06^a,b^	<0.001
SBP (mmHg)	101.09 ± 0.84	103.48 ± 0.16	106.97 ± 0.45^a,b^	110.25 ± 0.65^a,b^	<0.001
DBP (mmHg)	65.75 ± 0.75	65.68 ± 0.14	66.22 ± 0.40^a,b^	69.12 ± 0.58^a,b^	<0.001
Glucose (mg/dL)	89.19 ± 0.66	88.56 ± 0.12	89.67 ± 0.35^a,b^	90.74 ± 0.51^a,b^	<0.001
T-C (mg/dL)	158.95 ± 2.45	162.99 ± 0.46	168.47 ± 1.30^a,b^	170.88 ± 1.88^a,b^	<0.001
TGs	76.97 ± 4.48	82.20 ± 2.37	102.53 ± 2.37^a,b^	117.91 ± 3.43^a,b^	<0.001
HDL-C	55.61 ± 0.95	53.42 ± 0.17	48.63 ± 0.49^a,b^	45.82 ± 0.70^a,b^	<0.001
LDL-C	87.94 ± 2.15	93.09 ± 0.40	99.28 ± 0.14^a,b^	101.42 ± 1.65^a,b^	<0.001

Data are presented as the mean ± standard error (SE).

TMI, tri-ponderal mass index; WC, waist circumference; SBP, systolic blood pressure; DBP, diastolic blood pressure; T-C, total cholesterol; HDL-C, high-density lipoprotein cholesterol; TGs, triglycerides; LDL-C, low-density lipoprotein cholesterol.

Adjusted mean values of cardiometabolic risk factors were determined after controlling for age, alcohol consumption, smoking, household income, physical activity, rural residence, hypertension, diabetes mellitus, and dyslipidemia using an analysis of covariance (ANCOVA) according to sex-specific tri-ponderal mass index (TMI) groups.

a; P < 0.05 vs underweight group.

b; P < 0.05 vs normal weight group.

c: P < 0.05 vs overweight group.

### Adjusted ORs of MetS and its components according to sex- and age-specific TMI obesity groups

**Figure 2:**
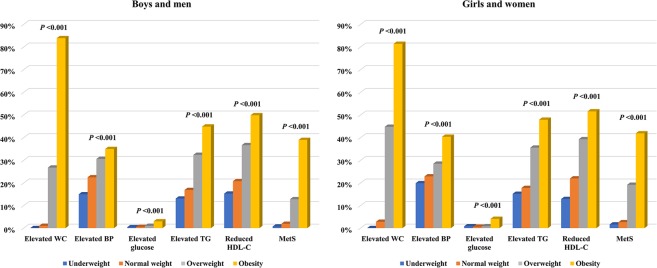
The prevalence of MetS and its components according to sex- and age-specific tri-ponderal mass index (TMI) groups (n = 8,464). Statistical significance was determined using chi-square tests. WC, waist circumference; BP, blood pressure; TGs, triglycerides; HDL-C, high-density lipoprotein cholesterol; MetS, metabolic syndrome.

The prevalence of MetS and its components are shown in Fig. 2. Among boys and men, the prevalence of elevated WC, elevated BP, elevated glucose, elevated TGs, reduced HDL-C, and MetS was 0%, 15.0%, 0.4%, 13.1%, 15.3%, and 0.7% in the underweight group; 1.0%, 22.5%, 0.5%, 16.9%, 20.8%, and 1.9% in the normal weight group; 26.8%, 30.6%, 1.0%, 32.4%, 36.7%, and 12.8% in the overweight group; and 83.9% (*P* < 0.001), 35.0% (*P* < 0.001), 3.0% (*P* < 0.001), 44.9% (*P* < 0.001), 49.9% (*P* < 0.001), and 39.0% (*P* < 0.001) in the obesity group according to the sex- and age-specific TMI groups, respectively. Among girls and women, the prevalence of elevated WC, elevated BP, elevated glucose, elevated TGs, reduced HDL-C, and MetS was 0%, 19.9%, 0.8%, 15.2%, 12.9%, and 1.6% in the underweight group; 2.8%, 22.9%, 0.6%, 17.8%, 22.0%, and 2.6% in the normal weight group; 44.8%, 28.5%, 0.8%, 35.6%, 39.3%, and 19.2% in the overweight group; and 81.4% (*P* < 0.001), 40.4% (*P* < 0.001), 4.1% (*P* < 0.001), 47.9% (*P* < 0.001), 51.6% (*P* < 0.001), and 41.9% (*P* < 0.001) in the obesity group according to sex- and age-specific TMI groups, respectively.

The adjusted odds ratios (ORs) for elevated WC, elevated BP, elevated glucose, elevated TGs, reduced HDL-C, and MetS are presented in Table 4. Compared to those in the normal weight group, the subjects in the overweight group exhibited an increased OR (29.18 (95% confidence interval (CI), 23.93–35.59)) for elevated WC, elevated BP (1.33 (1.13–1.56)), elevated TGs (2.55 (2.18–2.97)), reduced HDL-C (2.31 (1.99–2.68)), and MetS (8.93 (7.12–11.20)). The participants in the obesity group had increased ORs for elevated WC (154.67 (115.81–206.56)), elevated BP (2.22 (1.81–2.71)), elevated glucose (3.54 (1.56–8.06)), elevated TGs (4.12 (3.36–5.06)), reduced HDL-C (3.69 (3.01–4.52)), and MetS (25.57 (19.92–32.82)) compared to those of the participants in the normal weight group.

**Table 4:** Adjusted odds ratios (ORs) and 95% confidence intervals (CIs) of metabolic syndrome (MetS) and its components according to sex- and age-specific tri-ponderal mass index (TMI) groups (*n* = 8,464).

All participants					
	TMI group				
	Underweight	Normal weight	Overweight	Obesity	*P* for trend
Elevated WC^a^	NA	Reference	29.18 (23.93–35.59)	154.67 (115.81–206.56)	<0.001
Elevated BP^a^	0.82 (0.61–1.12)	Reference	1.33 (1.13–1.56)	2.22 (1.81–2.71)	<0.001
Elevated glucose^1^	0.77 (0.10–5.64)	Reference	1.41 (0.59–3.37)	3.54 (1.56–8.06)	0.004
Elevated TGs^a^	0.82 (0.58–1.17)	Reference	2.55 (2.18–2.97)	4.12 (3.36–5.06)	<0.001
Reduced HDL-C^a^	0.52 (0.36–0.76)	Reference	2.31 (1.99–2.68)	3.69 (3.01–4.52)	<0.001
MetS^a^	0.54 (0.20–1.51)	Reference	8.93 (7.12–11.20)	25.57 (19.92–32.82)	0.001
**Boys and men**					
	**Underweight**	**Normal weight**	**Overweight**	**Obesity**	
Elevated WC^b^	NA	Reference	41.48 (30.94–55–62)	240.88 (155.46–373.24)	<0.001
Elevated BP^b^	0.93 (0.62–1.39)	Reference	1.54 (1.25–1.91)	2.42 (1.82–3.23)	<0.001
Elevated glucose^b^	NA	Reference	0.70 (0.16–2.99)	1.48 (0.35–6.32)	0.582
Elevated TGs^b^	0.83 (0.52–1.34)	Reference	2.95 (2.39–3.64)	4.58 (3.44–6.09)	<0.001
Reduced HDL-C^b^	0.61 (0.39–0.95)	Reference	2.41 (1.97–2.96)	3.30 (2.48–4.39)	<0.001
MetS^b^	0.89 (0.32–2.45)	Reference	9.11 (6.80–12.19)	25.37 (18.10–35.57)	<0.001
**Girls and women**					
	**Underweight**	**Normal weight**	**Overweight**	**Obesity**	
Elevated WC^b^	NA	Reference	21.11 (16.03–27.80)	107.50 (72.68–159.00)	<0.001
Elevated BP^b^	0.67 (0.40–1.12)	Reference	1.10 (0.86–1.41)	2.07 (1.52–2.82)	<0.001
Elevated glucose^b^	2.89 (0.37–22.74)	Reference	2.97 (0.94–9.42)	8.04 (2.75–12.52)	<0.001
Elevated TGs^b^	0.84 (0.50–1.41)	Reference	2.19 (1.74–2.75)	3.84 (2.85–5.17)	<0.001
Reduced HDL-C^b^	0.35 (0.17–0.73)	Reference	2.29 (1.83–2.88)	4.54 (3.38–6.10)	<0.001
MetS^b^	NA	Reference	9.26 (6.38–13.43)	30.36 (20.58–44.79)	<0.001

TMI, tri-ponderal mass index; WC, waist circumference; BP, blood pressure; NA, not applicable; HDL-C, high-density lipoprotein cholesterol; TGs, triglycerides; MetS, metabolic syndrome.

^a^Adjusted odds ratios (ORs) of metabolic syndrome (MetS) and its components were determined after controlling for age, sex, alcohol consumption, smoking, household income, physical activity, rural residence, hypertension, diabetes mellitus, and dyslipidemia using a multiple logistic regression analysis according to sex- and age-specific tri-ponderal mass index (TMI) groups.

^b^Adjusted odds ratios (ORs) of metabolic syndrome (MetS) and its components were determined after controlling for age, alcohol consumption, smoking, household income, physical activity, rural residence, hypertension, diabetes mellitus, and dyslipidemia using a multiple logistic regression analysis according to sex- and age-specific TMI groups.

In the subgroup analyses, boys and men in the overweight group exhibited increased ORs for elevated WC (41.48 (30.94–55.62)), elevated BP (1.54 (1.25–1.91)), elevated TGs (2.95 (2.39–3.64)), reduced HDL-C (2.41 (1.97–2.96)), and MetS (9.11 (6.80–12.19)) compared to those of the participants in the normal weight group. Compared to those in the normal weight group, the boys and men in the obesity group had increased ORs for elevated WC (240.88 (155.46–373.24)), elevated BP (2.42 (1.82–3.23)), elevated TGs (4.58 (3.44–6.09)), reduced HDL-C (3.30 (2.48–4.39)), and MetS (25.37 (18.10–35.57)). Compared to those in the normal weight group, female subjects in the overweight group exhibited increased ORs for elevated WC (21.11 (16.03–27.80)), elevated TGs (2.19 (1.74–2.75)), reduced HDL-C (2.29 (1.83–2.88)), and MetS (9.26 (6.38–13.43)). The girls and women in the obesity group had increased ORs for elevated WC (107.50 (72.68–159.00)), elevated BP (2.07 (1.52–2.82)), elevated glucose (8.04 (2.75–12.52)), elevated TGs (3.84 (2.85–5.17)), reduced HDL-C (4.54 (3.38–6.10)), and MetS 30.36 (20.58–44.79) compared to those of the participants in the normal weight group.

## Discussion

This nationally representative population-based study showed the distribution of TMI according to sex and age, which was apparently stable compared to that of BMI. A covariance analysis revealed that obesity groups categorized according to sex- and age-specific TMI were positively correlated with WC SDS; SBP; DBP; and levels of glucose, T-C, and LDL-C but were inversely correlated with HDL-C levels in both sexes. Multiple logistic regression analyses revealed increased ORs for elevated WC, elevated BP, elevated TGs, reduced HDL-C, and MetS in subjects in the overweight group categorized according to sex- and age-specific TMI compared to those of the participants in the normal weight group categorized according to TMI among all participants. Higher ORs for elevated WC, elevated BP, elevated glucose, elevated TGs, reduced HDL-C, and MetS were observed in subjects in the obesity group categorized according to sex- and age-specific TMI compared to the normal weight group among all subjects using multiple logistic regression analyses.

With regard to diagnosing pediatric obesity, the weight-to-(height)^n^ ratio (kg/(m)^n^) has been suggested to explicate the effects of children’s growth during puberty, and the adjustment for value of n is decisive because inaccurate values lead to classifying tall or physically advanced children as overweight or obese^11,12^. An n value of two, which is known as BMI, provides precise information when height is constant as seen in adults. In pre-school children, BMI could be an adequate method for determining adiposity^11^. However, during puberty, changes in height increase the n value^12^. During the peripubertal phase, adiposity increases as growth and development progresses^13,14^. The value of n gradually increases from two to three; children who experience a rapid growth due to pubertal development have a tendency to have higher adiposity compared to less mature children exhibiting the same height^11^. It is necessary to increase values of n in physically advanced children who experience weight gain because higher BMI values are due to rapid increases in weight during pubertal development. Accordingly, BMI (value of n = 2) may be related to the overdiagnosis of children and adolescents with overweight and obesity^15^. Nevertheless, to date, BMI has been an important and recommended tool worldwide that was been used to identify children and adolescents with overweight or obesity. The guidelines of the Pediatric Endocrine Society and European Society of Endocrinology recommend the diagnosis of overweight or obesity in children and adolescents ≥ 2 years of age using BMI^16^. BMI values cannot be used directly for the identification of obesity, but BMI SDS or BMI percentile could be used in children and adolescents. Studies are being conducted to identify more reliable and more intuitive tools for clinicians, patients, and parents. The TMI (value of n = 3) has been suggested as a more accurate measure of body fatness to overcome some of the limitations associated with BMI. Peterson *et al*.^8^ suggested that the relative stability of TMI values according to sex and age may overcome the inconvenience of interpreting obesity based on BMI values during this period. In their study, TMI was a better estimate of body fat than the BMI SDS, and the R^2^ of TMI and BMI was 0.64 and 0.38 in boys and men and 0.72 and 0.66 in girls and women^8^, respectively. In addition, a very recent Canadian study demonstrated the distribution of TMI according to sex and age using the LMS method^10^. In Canadian children and adolescents, the TMI values were fairly stable compared to BMI values^10^. The present study is consistent with previous studies. In our study, the distribution of TMI was fairly stable compared to that of BMI according to the 2017 Korean national growth chart^17^.

TMI, which is a simple formula of anthropometrical measurements, may provide more accurate information reflecting body composition. Dual-energy X-ray absorptiometry (DXA), which is considered the gold standard for measuring body composition, is not always applicable in the clinical setting^18^. It is notable that greater amounts of abdominal fat were significantly associated with cardiometabolic risk factors in childhood, independent of BMI^19^. Therefore, TMI may provide more accurate information for children and adolescents with obesity and its complications who require early identification and intervention, with the advantage of simple anthropometrical measurements and intuitive interpretation in the primary clinical setting with no requirement of specific instruments, including DXA. On the other hand, potential limitations should be considered in the clinical application of anthropometrical measurements, including BMI and TMI. Both BMI and TMI do not fully differentiate fat mass from muscle mass^20^. With regard to the relationship between BMI and cardiometabolic risk factors, a series of studies were conducted in the past few decades. BMI and BMI SDS have also been demonstrated to be significantly relevant anthropometric markers to predict metabolic risk in youth^21–23^. However, few studies have been conducted regarding the association of TMI with cardiometabolic risk factors^10^. The present study showed that obesity groups categorized according to sex- and age-specific TMI are strongly correlated with cardiometabolic risk factors in covariance analysis and multiple logistic regression analysis. Our results could support the clinical application of TMI in the pediatric field. Future studies should be conducted to validate the use of TMI in the clinical setting.

TMI is considered to offer certain or fixed cutoff values because the distribution of TMI was fairly stable in previous studies^8,10^. Clinicians may identify obese children and adolescents with obesity and its complications who need early intervention during height and weight development and even during rapid growth using a certain TMI value, rather than using a BMI percentile, with convenience in a clinical setting. In a Spanish study, the mean TMI values according to sex and age were considerably stable, ranging from 12.2 kg/m^3^ to 12.7 kg/m^3^ in males and from 11.9 kg/m^3^ to 12.7 kg/m^3^ in females, respectively^24^. The distribution of the 50th percentile values of sex- and age-specific TMI was also fairly stable in a Canadian study^10^. However, the distribution of the 85th and 95th percentiles of sex- and age-specific TMI, which is the borderline of overweight and obesity, is considered not to be stable compared to that of the 50th percentile of sex- and age-specific TMI. In a recent study by Ashley-Martin *et al*., the range of the 97th percentile was reported to be approximately 18.5 kg/m^3^ to 20.5 kg/m^3^ in boys and men aged 10–20 years and approximately 18.5 kg/m^3^ to 23.0 kg/m^3^ in girls and women aged 10–20 years^10^. In the current study, the range of the 95th percentile, which is borderline obesity, presented from 16.82 kg/m^3^ to 17.40 kg/m^3^ in male subjects and from 16.00 kg/m^3^ to 18.06 kg/m^3^ in female participants. It is considered that a certain cutoff point of TMI would not be applicable for defining obesity in children and adolescents based on previous and current studies. Sex- and age-specific TMI could be used. The present study showed that obesity groups according to sex- and age-specific TMI are related to cardiometabolic risk factors in ANCOVA and multiple logistic regression after adjusting for possible confounders. Our study could provide valuable information.

The present study permits some limitations. First, we analyzed data using a cross-sectional nature, so causality between obesity according to sex- and age-specific TMI and obesity and cardiometabolic risk factors could not be determined. Second, the distribution of TMI could not be analyzed using longitudinal data. Caution should be used when interpreting the distribution of TMI because growing children and adolescents may move up or down across the reference percentile in height or weight. Third, we could not perform the statistical analysis using levels of sex hormones or Tanner stage, which may affect the scaling of weight with height^11^. Unexpectedly, we could not find a significant relationship between elevated glucose and the obesity group categorized according to TMI in boys and men after adjustment for confounders, whereas this relationship was significant with no adjustment. It may be related to relatively small population with elevated glucose compared to other components of MetS. Also, sex differences and age may be related. A recent study revealed that the mean HbA1c was higher in male youth aged 10–19 years (compared to young adult males aged 20–29 years) independent of obesity^25^. This finding may weaken the relationship between TMI obesity groups and elevated glucose in our analysis among boys and men but not girls. Further studies should be conducted with populations of different ethnicities and various ages.

In conclusion, this population-based study demonstrated the distribution of TMI according to sex and age. The distribution of the median and mean TMI values according to sex and age showed a considerably stable nature compared to that of BMI. Obesity groups according to sex- and age-specific TMI were positively correlated with WC SDS; SBP; DBP; and levels of glucose, T-C, TGs, and LDL-C but were negatively correlated with HDL-C in both sexes using ANCOVA after adjusting for confounding factors. In multiple logistic regression, higher ORs for elevated WC, elevated BP, elevated TGs, reduced HDL-C, and MetS were observed in participants in the overweight groups than in those in the normal weight group categorized according to sex- and age-specific TMI. Children and adolescents in the obesity group according to sex- and age-specific TMI had increased ORs for elevated WC, elevated BP, elevated glucose, elevated TGs, reduced HDL-C, and MetS compared to those of participants in the normal weight group after adjusting for confounders. These findings suggest that sex- and age-specific TMI may be applicable in the clinical setting as a useful screening tool for children and adolescents with overweight and obesity and that sex- and age-specific TMI has the advantages of an apparently stable distribution and considerably easy interpretation.

## Materials and Methods

### Study population

**Figure 3:**
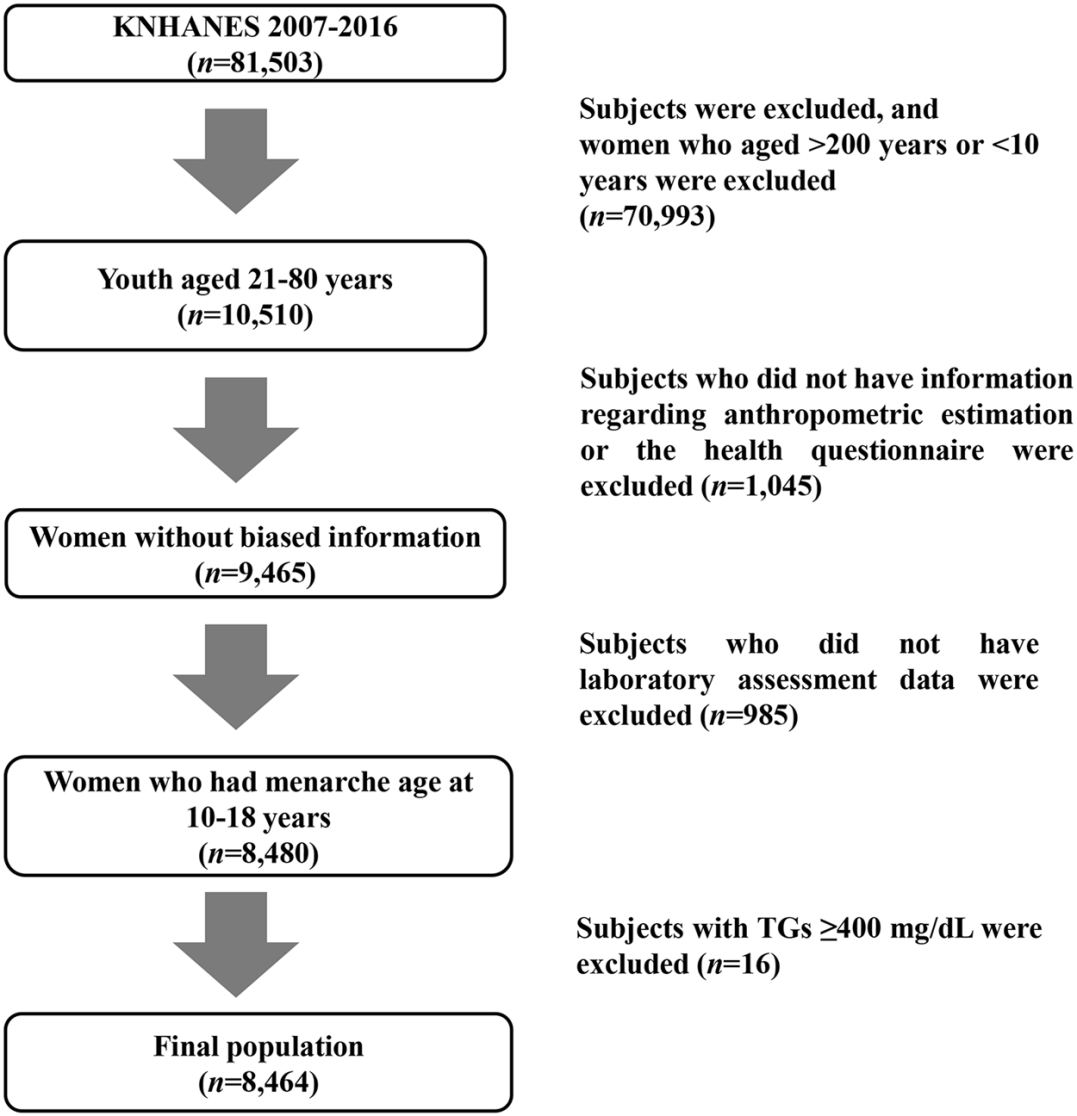
Flow chart of study population.

We conducted statistical analyses using the dataset from the Korea National Health and Nutrition Examination Survey (KNHANES) for the period 2007–2016 in this study. The KNHANES, a population-based cross-sectional survey, is designed to assess the state of health and nutrition in a nationally representative sample of noninstitutionalized Korean individuals^26^. The survey selects participants as household units using a stratified and multistage probability sampling method. The Korean national survey comprises three parts: (i) health questionnaire, (ii) health examination, and (iii) nutritional assessment. Previous studies have described details of the KNHANES^26,27^. The KNHANES 2007–2016 enrolled a total of 81,503 subjects. Of these participants, we included 10,510 youth aged 10–20 years in preliminary analyses. We excluded males and females who did not have information regarding anthropometric estimation or the health questionnaire (*n* = 1,045). We excluded subjects who did not have laboratory assessment data (*n* = 985). We also excluded participants with TGs ≥ 400 mg/dL (*n* = 16), because we determined LDL-C using Friedewald’s equation^28^. Finally, we analyzed 8,464 participants aged 10–20 years who had complete information from KNHANES 2007–2016 in this study (Fig. 3). All datasets from KNHANES are accessible for the public at the website (http://knhanes.cdc.go.kr). The study protocol for KNHANES 2007–2016 was approved by the Institutional Review Boards of the Korean Centers for Disease Control and Prevention. Informed consent was obtained from all KNHANES participants and their parents or legal guardians. All methods in KNHANES were conducted in accordance with relevant guidelines and regulations. The present study was also approved by the Institutional Review Board of the Hallym University Dongtan Sacred Heart Hospital (IRB No. 2019–08–015).

### Measurements

The determination of height, weight, WC, SBP and DBP was performed in accordance with standard methods. BMI and TMI were estimated as the ratios of weight to height squared (kg/m^2^) and weight to height cubed (kg/m^3^), respectively. The SDS values for height, weight, WC and BMI were assessed using LMS methods based on the 2017 Korean national reference^17^. SBP and DBP (mmHg) were assessed from the right upper arm three times using a calibrated sphygmomanometer and an appropriately sized cuff. Respective determination was conducted at intervals of 2 minutes. The mean SBP and DBP of the last two assessments were used for analysis.

Blood samples were obtained at the lateral aspect of forearm year-round after participants had fasted for more than 8 hours. The collected blood samples were analyzed in a central laboratory (NeoDin Medical Institute, Seoul, Korea) within 24 hours. The biochemical analyses of glucose, T-C, HDL-C, and TGs were performed using an automatic analyzer (Hitachi 7600, Hitachi, Tokyo, Japan). The level of LDL-C (mg/dL) was determined based on Friedewald’s method^28^.

### Collection of lifestyle parameters

The lifestyle variables were evaluated using alcohol consumption, smoking, household income, physical activity, and residence. Alcohol consumers were divided into two groups (consumers vs. nonconsumers). Smokers were classified into two groups (smokers vs. nonsmokers). Physical activity was classified into the two groups (exercise or no exercise). Exercise was defined as meeting one or more of the following criteria: (i) vigorous activity for greater than or equal to 20 minutes/day and 3 days/week or (ii) moderate activity or walking for greater than or equal to 30 minutes/day and 5 days/week. The place of residence was classified into two groups (urban vs. rural).

### Definition

MetS and its components were defined according to modified pediatric and adolescent criteria of the National Cholesterol Education Program III (NCEP III)^29^. Elevated WC was defined as a sex- and age-specific WC equal to or greater than the 90th percentile. Elevated BP was defined as sex-, age-, and height-specific SBP or DBP equal to or greater than the 90th percentile based on the 2017 Korean reference^17^ or when subjects were receiving treatment for hypertension. Elevated glucose was defined as levels of glucose equal to or greater than 110 mg/dL in fasting state or when subjects were previously diagnosed with T2DM. Participants with a previous diagnosis of type 1 diabetes mellitus (T1DM) were excluded from this study. T2DM was defined as one or more of the following criteria: (i) self-reported T2DM in questionnaire during the survey, (ii) current administration of oral antidiabetic medication and/or subcutaneous insulin for managing T2DM, or (iii) level of glucose equal to or greater than 126 mg/dL at fasting state during survey. Elevated TGs were defined as a level of TGs equal to or greater than 110 mg/dL at fasting state or when subjects were receiving treatment for dyslipidemia. Reduced HDL-C was defined as HDL-C levels less than 40 mg/dL. MetS was defined as one or more of following criteria: (i) elevated WC, (ii) elevated BP, (iii) elevated glucose, (iv) elevated TGs, and (v) reduced HDL-C.

### Statistical analyses

R version 3.6.1 for Windows (The R Foundation for Statistical Computing, Vienna, Austria) was used for statistical analyses. The participants in the present study were classified into four groups according to sex- and age-specific TMI: (i) underweight group: TMI < 3rd percentile, (ii) normal weight group: TMI ≥ 3rd percentile and < 85th percentile, (iii) overweight group: TMI ≥ 85th percentile and < 95th percentile, and (iv) obesity group: TMI ≥ 95th percentile. To determine statistical significance, analysis of variance (ANOVA) and chi-square tests were used for continuous variables and categorical variables according to sex- and age-specific TMI obesity groups. Continuous variables and categorical variables are presented as the means ± standard deviations (SDs) and the percentages (%). Adjusted means for cardiometabolic risk factors, including WC SDS; SBP; DBP; and levels of glucose, T-C, HDL-C, TGs, and LDL-C were estimated using ANCOVA with Tukey’s *post hoc* test after controlling for possible confounders including age, alcohol consumption, smoking, household income, physical activity, rural residence, hypertension, DM, and dyslipidemia according to sex- and age-specific TMI obesity groups. To investigate the relationship of sex- and age-specific TMI groups with MetS and its components, a multiple logistic regression analysis was conducted. In model 1, the adjusted ORs and 95% CIs for MetS and its components were determined after adjustment for age, sex, alcohol consumption, smoking, household income, physical activity, rural residence, hypertension, DM, and dyslipidemia according to sex- and age-specific TMI obesity groups among all subjects. In model 2 of the multiple logistic regression analysis, the adjusted ORs (95% CIs) for MetS and its components were assessed after adjusting for age, alcohol consumption, smoking, household income, physical activity, rural residence, hypertension, DM, and dyslipidemia according to sex-specific TMI obesity groups both in boys and men and in girls and women. The adjusted ORs (95% CIs) for MetS and its components were determined with the normal weight group serving as a reference. Statistical significance was determined when the *P* value was <0.05 using a two-tailed method.
